# Effects of a four-year health systems intervention on the use of maternal and infant health services: results from a programme evaluation in two districts of rural Chad

**DOI:** 10.1186/s12889-021-12330-2

**Published:** 2021-12-19

**Authors:** Séverine Erismann, Jean-Pierre Gami, Boukari Ouedraogo, Damien Revault, Helen Prytherch, Filippo Lechthaler

**Affiliations:** 1 Swiss Tropical and Public Health Institute, P.O. Box, CH-4002 Basel, Switzerland; 2 University of Basel, P.O. Box, CH-4003 Basel, Switzerland; 3 Centre de Support en Santé Internationale, N’Djamena, Chad; 4 School of Agricultural, Forest and Food Sciences, Bern University of Applied Sciences, Zollikofen, Canton of Bern Switzerland

**Keywords:** Antenatal care, Maternal and child care, Quality of care, Health system strengthening, Low resource, Rural setting

## Abstract

**Background:**

Attendance of maternal and infant care services in rural Chad are consistently low. Our study aimed to assess the use of antenatal (ANC) and postnatal care (PNC) services, health facility delivery and infant health services after 4 years of a health systems intervention for improving the infrastructure, supplies, training and sensitization for maternal and infant health in two districts of rural Chad.

**Methods:**

Data from a repeated cross-sectional household survey conducted in Yao and Danamadji in 2015 and in 2018 were analyzed. A stratified two-stage cluster sampling methodology was applied to achieve a representative sample of the rural settled and mobile population groups in the study area. A generalized linear model was applied to determine the health care utilization rates. Multivariate regression models were used to assess the association between the programme intervention and utilization outcomes of selected maternal and infant health services.

**Results:**

Complete datasets were available for 1284 households at baseline. The endline analysis included 1175 households with complete survey data. The use of at least one ANC amongst pregnant women increased in both settled communities (from 80% in 2015 to 90% in 2018) and amongst mobile pastoralist communities (from 48% in 2015 to 56% in 2018). The rate of home delivery among settled communities and mobile pastoralists changed little between baseline and endline and remained high for both population groups. Individuals that were covered by the health systems intervention were however significantly more likely to attend ANC and less likely to give birth at home. PNC services only showed improvements amongst the settled communities (of 30%). Infants’ reported health outcomes and vaccination coverage considerably improved; the latter especially among mobile pastoralist (from 15% in 2015 to 84% in 2018).

**Conclusion:**

A combination of health systems strengthening interventions was associated with an increased use of certain maternal and infant health services. However, to facilitate equitable access to and use of health care services in particular in times of increased vulnerability and by certain population groups in hard-to-reach areas, reinforced health education and culturally adapted communication strategies, including gender-specific messaging will be needed over a sustained period.

**Supplementary Information:**

The online version contains supplementary material available at 10.1186/s12889-021-12330-2.

## Background

In Chad, maternal mortality rates are amongst the highest worldwide at 860 per 100,000 live birth in 2015. One in 16 women dies due to complications while giving birth, which translates to a rate of 6.2%. Only 22% of women are assisted by qualified health personal while giving birth in 2015 [1]. This figure is likely to be much lower in rural areas and particularly for mobile pastoralists, due to factors like geographical inaccessibility of modern medicine, political neglect, and cultural preferences, exacerbating their vulnerabilities [2].

Mirroring high maternal health mortality rates [3], child mortality rates lie at 12.5% in 2015 [1]. Most deaths of children under the age of five are due to all sorts of diseases (66%), while 43% are attributable to malnutrition [4]. These occur primarily in the context of lack of preventive and primary health care, including maternal and child health care [3, 5]. In 2015, only 25% of infants aged 12–23 months were completely vaccinated against targeted childhood diseases, which exemplifies the low preventive child consultation rate [1, 3].

Chad’s health system is chronically weak [3, 6], as indicators of infant and maternal mortality and the prevalence of endemic and epidemic diseases show. Foremost among them are malaria, tuberculosis, acute respiratory infections, the human immunodeficiency virus (HIV) and acquired immunodeficiency syndrome (AIDS) and diarrhea [1]. Health spending has constituted 3.1% of its Gross Domestic Product as of 2016 [7]. Chad’s health system is not only chronically underfunded but also characterized by poor quality of care [3] – which is also known to influence the trust that people are ready to place in health services [8]. Insufficient qualified health personnel, very irregular supplies of inputs (medicines and consumables), and hence, a low utilization of primary health care facilities are further attributes of poor quality health services in Chad [3, 9]. In consequence, the health status of the population is compromised with high levels of disease and malnutrition, which interacts negatively with poor quality of care to make health outcomes even worse.

To support the government to address some of these challenges and in strengthening its health system, the Swiss Tropical and Public Health Institute (Swiss TPH) has implemented together with the Centre de Support en Santé Internationale (CSSI) a collaborative health system intervention programme for improving maternal and new-born health in two districts of rural Chad, Yao and Danamadji. The *Programme d’appui aux Districts Sanitaires* (PADS) was funded by the Swiss Agency for Development and Cooperation (SDC). It was launched in 2014 and ran until 2018 (first phase). The programme was implemented by a country team in N’Djamena and in each of the two supported health districts. As part of the PADS project, a baseline and endline study were conducted in the two districts Yao and Danamadji. Here, we report findings on the associations between the PADS interventions and the use of maternal and infant health services.

## Methods

### Study setting

The baseline and endline study were conducted in the two same health districts, namely in Yao (Batha region) and Danamadji (Moyen-Chari region). The study side and its characteristics have been described in the baseline manuscript [10]: health service delivery is mainly provided through primary health centers, while each district has one hospital acting as secondary referral center. Furthermore, the district is seasonally populated by a substantial number of mobile pastoralist communities (mainly camel and cattle breeders of the Arab and Fulani pastoralist ethnic groups) In the dry season, mobile communities are often clustered in smaller and larger camps composed of several families in a more stable way around concentration zones near water points [10].

### Study design and sampling method

The present analysis is based on a repeated cross-sectional approach building on two random samples in different time periods (baseline and endline). The study was originally designed as a cross-sectional baseline survey [10], where the study design is described in more detail. The STROBE checklist was applied as an internal quality control measure for the use of this design. In summary, referring to [10], the survey included households with an adult mother of a child younger than 5 years. The sample size calculation aimed at obtaining a given precision of ANC utilization rates for settled communities and mobile pastoralists assuming an average prevalence of 25%, a standard error of +/− 0.025 and an intra-class correlation coefficient of 0.1. The same sampling approach has been applied for both waves of cross-sectional surveys. In total, for each survey, 786 mothers from rural settled communities and 358 mothers from mobile pastoralist communities were randomly sampled.

**Fig. 1:**
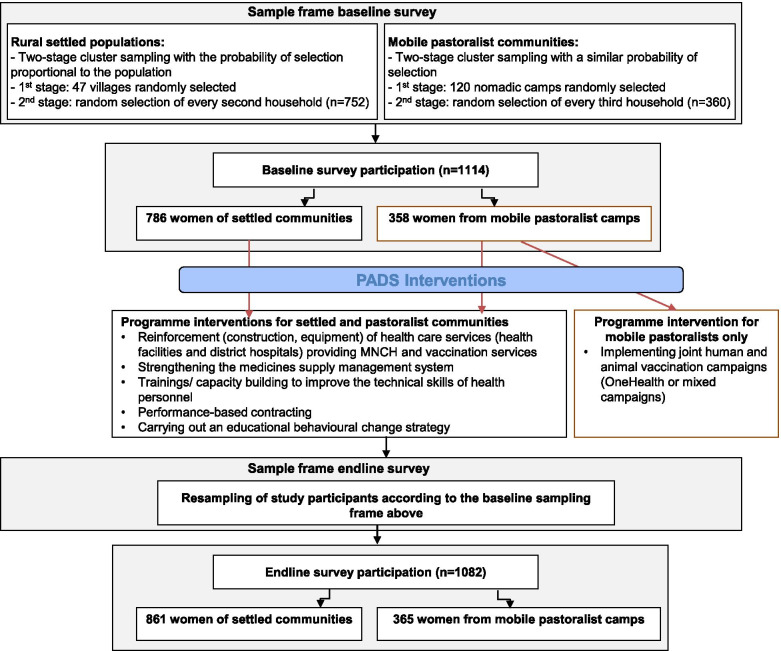
Visualization of the stratified two-stage cluster sampling methodology. Description of data: The figure shows the stratified two-stage cluster sampling methodology applied to achieve a representative sample of the two population groups in the study area

The applied stratified two-stage cluster sampling methodology is describe in detail in [10] and visualized in Fig. 1. For rural settled populations, the sampling strategy was based on villages (first stage) and households (second stage). The same selection of the 47 villages was used for the baseline and endline survey. Households were re-sampled during the second wave. For mobile pastoralist community, the sampling strategy was based on nomadic camps (first stage) and households (second stage). The same selection of 120 camps was used for the baseline and endline survey. Households were re-sampled during the second wave.

### Data collection

Training and all field activities were overseen by the study investigators. All interviewers spoke the local language. The baseline survey was conducted between May and June in 2015, and the endline survey was conducted between June and July in 2018. The same field procedures were used in the baseline and endline surveys, which have been described in detail elsewhere [10].

The same structured questionnaire that had been developed at baseline in French, specifically for this study, was administered. It covered the use of maternal and infant health services and sociodemographic characteristics was administered in households using tablets (Samsung Galaxy note 10.1 N8010) and open data kit (ODK) software [11].

### Analysis

Frequencies and proportions were used for the descriptive analysis analyzing both random samples separately (baseline and endline). A generalized linear (binomial) model was applied to determine the utilization rates of care services with a 95% confidence interval. Clusters (camps or villages) were taken into account as a random effect. An asset index was created applying principal component analysis to household responses on possession of assets [12]. A multivariate generalized linear regression model with binomial link function was used to assess several factors that best predict health service utilization rates based on a pooled cross section combining the two random samples. Outcome variables were ANC1, home delivery, PNC with infant and vaccination. The predictors used were: year of the survey (2015 and 2018), level of education (none, primary, secondary or more), socio-economic status (according to the asset index), population group affiliation (settled or mobile), gender, district (regional location), and having been affected through a PADS intervention. To facilitate interpretation of the estimated coefficients, regression results were transformed and interpreted using the concepts of odds-ratios. More specifically, for each coefficient a odds-ration was calculated through the usual exponentiation operation [13]. All variables used were obtained through the repeated cross-sectional design using the same structured-household survey during the baseline and endline survey. Health service utilization rates are self-reported by the participants. The PADS intervention was measured by asking the study participant whether she has been part of a programme intervention such as mixed vaccination campaigns or health promotion activities. Data analysis was conducted in R statistical software. For multivariate regression the package glmmML was used.

As this study did not include an experimental design, the associations found show a general tendency. Looking at the association between the PADS intervention and the different outcomes several confounders create potential distortions in the estimated parameters: health utilization rates and the likelihood of being affected by the intervention are both linked to demographic factors (gender, age, education etc.), to socio-economic factors (income, education), population group affiliation as well as year of the survey. In order to minimize the effect of confounding during statistical analysis, multivariate regression has been used controlling for a variety of the confounding factors.

### Projet d’Appui aux districts Sanitaires du Tchad, phase 1 (2014–2018)

Data were collected as part of the PADS project, a collaborative health system intervention programme for improving maternal and newborn health in two districts of rural Chad. This first phase of the project was implemented between November 2014 and October 2018. The two selected districts, Yao and Danamadji, constitute the intervention area of this health implementation project. The main interventions consisted of several components: (1) reinforcement of health care services (health facilities and district hospitals) through the construction and rehabilitation of the infrastructures and the provision of biomedical equipment; (2) strengthening the medicines supply management system; (3) trainings/ capacity building to improve the technical skills of health personnel (surgery, integrated management of childhood illness, application of national protocols) and the managerial capacities of health administration (supervision, planning and leadership skills); (4) performance-based contracting to finance health activities and provide remuneration based on performance (‘contrats d’objectifs et de moyen’, COM); (5) carrying out an educational behavioural change strategy with community participation; and (6) implementing joint human and animal vaccination campaigns (OneHealth or mixed campaigns), including awareness raising on the benefits of vaccines and disease control programmes (Fig. 1). These interventions provided to settled and mobile communities as part of PADS were implemented to strengthen the first two healthcare levels of Chad (including basic health units, district hospitals in the first level and regional hospitals in the second level) according to its three level healthcare pyramid [14].

## Results

### Socio-demographic characteristics of study participants

At baseline, complete datasets were available for 1144 women. The dataset at endline included 1082 women, amongst which 64% are from settled communities and 36% are mobile pastoralists (Table 1, Fig. 1). Sociodemographic and economic characteristics between the two groups at baseline and endline were similar and are shown in Table 1.

**Table 1:** Characteristics of the study participants in two districts of Chad

Socio-demographic characteristics	Baseline 2015	Endline 2018
	n (%)	n (%)
**Participation**		
Women with infant	1144 (100%)	1082 (100%)
Settled communities	786 (69%)	810 (69%)
Mobile pastoralists	358 (31%)	365 (31%)
**Age group women**		
16–20	252 (22%)	233 (22%)
21–30	534 (47%)	542 (50%)
31–40	303 (27%)	249 (23%)
40+	53 (4%)	58 (5%)
**Educational attainment: settled communities**^a^		
Primary school (completed)	324 (41%)	380 (53%)
Secondary school (completed)	129 (16%)	257 (36%)
Higher education + (completed)	9 (1%)	3 (< 1%)
**Educational attainment: mobile pastoralists**^a^		
Primary school (completed)	34 (9%)	3 (1%)
Secondary school (completed)	4 (1%)	0
Secondary school + (completed)	0	0

^a^ % calculated with respect to the corresponding sub-sample

### ANC use and home delivery

At the endline survey, an increase in the percentage of pregnant women among settled communities who had at least one ANC was found (from 80% in 2015 to 90% in 2018). A smaller improvement was found for mobile pastoralists where the ANC attendance rate remained however low at 56%. With regards to women attending four or more ANC visits, utilization rate increased from 26 to 34% for settled communities and from 8 to 14% for mobile pastoralists (Table 2).

**Table 2:** Changes in antenatal care attendance among pregnant women in two districts of Chad

		ANC1^a^ (95% CI)		ANC4^b^ (95% CI)	
		Baseline	Endline	Baseline	Endline
Settled communities	Global	**80%** (73–86%)	**90%** (67–97%)	**25%** (21–32%)	**34%** (27–42%)
	Yao	45% (38–53%)	59% (48–69%)	7% (3–15%)	14% (6–31%)
	Danamadji	94% (91–96%)	97% (94–98%)	46% (42–51%)	61% (50–71%)
Mobile pastoralists	Global	**48%** (41–55%)	**56%** (41–70%)	**8%** (3–21%)	**14%** (11–18%)
	Yao	27% (19–37%)	13% (0.01–76%)	4% (1–17%)	6% (3–10%)
	Danamadji	74% (55–87%)	72% (65–78%)	17% (6–40%)	22% (17–29%)

^a^*ANC1* Number of pregnant women who attended at least the first antenatal care consultation

^b^*ANC4* Number of pregnant women who attended four antenatal consultations

The main reasons cited by women at baseline and endline for not attending ANC in Yao were: (i) distance and (ii) the fact that it is not part of their habits. In Danamadji, these were the same two reasons, with the predominant reason being that it is not part of their habits. Interestingly, while the reported costs and quality of services played an important role at baseline, these factors were given less importance at endline (Table 3).

**Table 3:** Changes in the reasons given by women for not attending the first ANC in two districts of Chad

		Distance		Costs		Insufficient quality		Poor reception		Not part of their habit	
		2015	2018	2015	2018	2015	2018	2015	2018	2015	2018
**Settled communities**	Global	**39%**	**31%**	35%	20%	6%	1%	2%	1%	**39%**	**31%**
	Yao	**43%**	**31%**	34%	20%	6%	1%	2%	1%	**43%**	**31%**
	Danamadji	**9%**	**27%**	**48%**	**18%**	0%	0%	0%	0%	0%	**18%**
**Mobile pastoralists**	Global	**36%**	**33%**	19%	5%	3%	1%	2%	3%	**51%**	**53%**
	Yao	**39%**	**35%**	19%	3%	2%	1%	2%	0%	**56%**	**44%**
	Danamadji	**29%**	**27%**	20%	10%	6%	0%	2%	10%	**37%**	**56%**

*Note*: the percentages in this table are calculated as simple proportions representing the number of reported reasons to the number of participants not attending the first ANC

The rate of home delivery among settled communities has decreased from 87 to 73%, whereas for mobile pastoralist this number increased globally from 92 to 100% (Table 4). On the other hand, we noted an important increase in the percentage of women among settled communities who attended PNC within 3 months of delivery with or without infant. These trends were not detectable for mobile pastoralists (Tables 5 and 6).

**Table 4:** Changes in the percentage of women who delivered at home in two districts of Chad

Home delivery (95% CI)			
		**Baseline 2015**	**Endline 2018**
**Settled communities**	Global	**87%** (77–93%)	**73%** (62–83%)
	Yao	93% (80–98%)	83% (64–94%)
	Danamadji	61% (54–69%)	51% (43–59%)
**Mobile pastoralists**	Global	**92%** (81–97%)	**100%**
	Yao	97% (82–99%)	100%
	Danamadji	88% (83–92%)	100%

**Table 5:** Changes in the percentage of women attending postnatal consultation without an infant in two districts of Chad

Postnatal consultation without infant (95% CI)			
		**Baseline 2015**	**Endline 2018**
**Settled communities**	Global	**21%** (14–30%)	**54%** (47–59%)
	Yao	5% (2–15%)	25% (21–30%)
	Danamadji	51% (44–58%)	96% (57–99%)
**Mobile pastoralists**	Global	**16%** (10–25%)	**8%** (6–11%)
	Yao	17% (9–29%)	2% (0–4%)
	Danamadji	14% (6–22%)	15% (10–20%)

**Table 6:** Changes in the percentage of women attending postnatal consultation with their infant in two districts of Chad

Postnatal consultation with infant (95% CI)			
		**Baseline 2015**	**Endline 2018**
**Settled communities**	Global	**27%** (21–35%)	**57%** (50–64%)
	Yao	11% (6–20%)	28% (17–40%)
	Danamadji	59% (48–68%)	96% (57–99%)
**Mobile pastoralists**	Global	**9%** (4–18%)	**5%** (3–8%)
	Yao	6% (2–19%)	0%
	Danamadji	15% (6–32%)	9% (6–14%)

### Self-reported infant health outcomes

During the baseline study, most of the mothers surveyed (72% of settled communities and 84% of mobile pastoralists) reported that their children were sick in the last 6 months. This percentage decreased significantly at endline, where 52% of settled communities and 67% of mobile pastoralists reported illness of their children the 6 months preceding the survey. This change was most detectable amongst settled communities and mobile pastoralist in Yao (Table 7). Among mobile pastoralists in Danamadji, the percentage of women reporting their infant being sick remained very high with 81% at endline (Table 7).

**Table 7:** Changes in the percentage of women who reported their infant being sick in the last 6 months in two districts of Chad

Infant sickness (reported by the mother) (95% CI)			
		**Baseline 2015**	**Endline 2018**
**Settled communities**	Global	**72%** (67–77%)	**52%** (47–56%)
	Yao	**75%** (67–81%)	**42%** (35–49%)
	Danamadji	70% (60–78%)	62% (53–71%)
**Mobile pastoralists**	Global	**84%** (75–90%)	**67%** (50–81%)
	Yao	**89%** (76–95%)	**59%** (52–66%)
	Danamadji	80% (73–85%)	81% (33–97%)

### Vaccination coverage

The vaccination coverage based on parental recall of the last child vaccinated has considerably increased at endline amongst mobile pastoralists from 15% in 2015 to 84% in 2018, and especially amongst mobile pastoralists in Danamadji (1% in 2015 to 85% in 2018). Among settled communities, a minor increase was detectable from 80% at baseline in 2015 and to 83% at endline in 2018 (Table 8).

**Table 8:** Changes in the percentage of vaccination coverage (parental recall) in two districts of Chad

		Vaccination coverage (95% IC)	
		Baseline 2015	Endline 2018
Settled communities	Global (*n* = 784)	**80%** (73–85%)	**83%** (70–91%)
	Yao (*n* = 415)	56% (50–62%)	67% (55–78%)
	Danamadji (*n* = 369)	93% (90–95%)	95% (92–97%)
Mobile pastoralists	Global (*n* = 358)	**15%** (7–30%)	**84%** (80–87%)
	Yao (*n* = 185)	18% (10–32%)	82% (76–87%)
	Danamadji (*n* = 173)	1% (0.1–19%)	85% (80–90%)

### Results from the multivariate analysis

The results of the multivariate analysis show a significant association between those who were in the coverage of a PADS intervention and the use of maternal health services. Individuals who reported to have received a PADS intervention were significantly more likely to attend at least one ANC and were less likely to give birth at home. This association was not observed for postnatal care. The results of the multivariate analysis also showed that individuals who were affected by a PADS intervention were much more likely to have their last child vaccinated (Table 9). Furthermore, the results indicate that settled population groups are significantly more likely to deliver at health facilities, use ANC and PNC services and vaccinate their children. Living in the district of Danamadji and having completed secondary school was also associated with higher utilization rates for all analyzed services. A high-income status measured through the socio-economic index was associated with a lower home delivery rate. The lower income status was associated with a lower PNC utilization.

**Table 9:** Association between health service use, vaccination coverage, and different socio-demographic and geographical variables

	ANC1	Home delivery	PNC^d^ with infant	Infant vaccinated
	OR^a^ (95% CI)	OR^a^ (95% CI)	OR^a^ (95% CI)	OR^a^ (95% CI)
Lower SES^b^	0.99 (0.78–1.26)	1.30 (1–1.70)	0.69 (0.55–0.88)**	1.11 (0.78–1.43)
Higher SES	0.79 (0.58–1.07)	0.72 (0.53–0.98)*	1.03 (0.76–1.39)	0.88 (0.64–1.20)
DS^c^ Danamadji	10.56 (8.12–13.71)**	0.18 (0.14–0.25)**	4.43 (3.49–5.63)**	5.56 (4.25–7.26)**
Settled population	4.22 (3.22–5.53)**	0.29 (0.22–0.41)**	4.38 (3.49–5.63)**	12.53 (8.80–17.89)**
Secondary school completed	3.80 (1.82–7.92)**	0.47 (0.34–0.67)**	4.45 (3.40–5.64)**	1.74 (1.20–2.52)**
Survey year 2018	1.02 (0.79–1.34)	0.58 (0.42–0.80)**	1.71 (1.33–2.20)**	1.63 (1.22–2.19)**
Affected by a PADS intervention	1.76 (1.26–2.45)**	0.66 (0.47–0.93)*	6.74 (4.56–9.96)**	0.85 (0.61–1.20)

*P < 0.05; **P < 0.01; ***P < 0.001

^a^Odds ratios (ORs) refer to the period effects. Multivariate linear regression models were adjusted for the categorical socio-economic status (SES) variable

^b^*SES* Socio-economic status

^c^District Sanitaire

^d^*PNC* Postnatal care

## Discussion

This evaluation reveals some progress in maternal and infant health service utilization in the two study districts. In particular, the results suggest an increase in the percentage of pregnant women among settled communities that attended ANC at least once, and that attended postnatal care within 3 months of delivery. Smaller improvements were found for mobile pastoralists, however, the ANC attendance rates remained low and PNC rates even decreased. The rate of home delivery remained also very high for both population groups. The vaccination coverage based on parental recall of their last child improved considerably amongst mobile pastoralists. Compared to the last national demographic and health survey (DHS) of 2014–2015, 55% of women attended at least one ANC (compared to 90% among settled communities and 56% among mobile pastoralists in this study). As for ANC3+, according to the last DHS, 31% attended at least three ANC, which is in line or higher than our findings with 34% for the settled communities and 14% for mobile pastoralists. However, these findings also point to some important challenges concerning interventions aiming to improve maternal and infant health outcomes. Healthcare during pregnancy is a priority. Poor antenatal attendance is associated with pregnancy complications, delivery of low birthweight babies and more newborn deaths [15]. The benefits of a pregnant woman attending ANC are also in terms of nutritional and health checks, such as whether a woman has a disease like malaria or has been exposed to other infectious diseases [16]. According to Mbuagbaw and Gofin [17], interventions to reduce maternal mortality may focus on three periods. The first is during pregnancy (ANC); the second is the intrapartum period, i.e. during labour and delivery, and the third is in the postpartum period (after delivery, PNC) [16]. The pregnancy period is the more stable period of these three, whilst the intrapartum period is much shorter and less predictable. Accordingly, it is often more challenging to provide universal care during this short period of labor and delivery, than in the longer and more stable ANC period [17].

The potential of ANC in improving maternal and neo-natal health has long been recognized since the 1990s [18]. In developing countries however, many pregnant women still fail to benefit from comprehensive ANC. Several factors have been identified for late initiation of ANC uptake, such as women’s education, husband’s education, women’s employment, affordability of services and access to the clinics [19], but also factors related to the poor quality of ANC services, such as the shortage of supplies and drugs and unskilled health personnel [20–22]. Although the findings of our study suggest that ANC attendance seem to have increased recently for settled communities mainly, our qualitative study shows that there remain serious issues with regards to quality of care and the availability of medicines [23], but also with regards to cultural factors (habits) and distance to primary health centers. This holds especially true for mobile pastoralists. Results from a scoping review on access to modern reproductive health services conducted amongst nomadic populations across the world highlighted that nomadic people face complex barriers to healthcare access, which were largely characterized as external (geographic isolation, socio-cultural dynamics, logistical and political factors) or internal (lifestyle, norms and practices, perceptions) factors. Furthermore, low awareness of modern reproductive and maternal health services and their benefits reinforced by a lack of culturally sensitive approaches to communicating about them, were mentioned as major barriers to utilization [24]. This is in line with the findings from our baseline survey and qualitative assessment, highlighting not only the importance of the availability, affordability, quality of care and distance to primary health centers but also of the health practitioners competencies in welcoming and cultural communication and the practices and customs of the target population [10, 25] – all of which interact to influence women’s likelihood to seek out or accept health services [26].

The emerging consensus is that in order to improve maternal and newborn health, a range of health system strengthening interventions at every level of the continuum of care, from the community to the health facility and hospital, are necessary, instead of fostering more traditional vertical programmatic strategies [27]. In these programmes, single elements of care are often implemented without making the needed connections to ensure comprehensive care [28]. Within the continuum of care, all women should have access to reproductive health choices and care during pregnancy and childbirth, and all newborns should be able to grow into healthy children [29]. This approach calls for the integration of programmes for maternal, neonatal, and child health that include a package of services including community-based family planning, health and nutrition services [29–31]. It has however also become increasingly evident that high coverage of essential interventions in healthcare facilities is not enough to reduce maternal mortality [28], mainly due to the services not being utilized [31]. Additionally and most importantly, women’s capacity and capability to take ownership and decide about their care at the right time needs to be strengthened. Too often, women are still dependent on others to make these decisions for them. According to Elmusharaf et al. [31], gender norms are often too little considered in the design, implementation and research of interventions and strategies to improve access to maternal healthcare. Unequal power in the decision-making process within households often restrict women’s autonomy, her negotiation power with her partner, increase fertility rates and unwanted pregnancy, and hence negatively affect maternal health outcomes [32]. Thus, in future more attention needs to be given to gender-specific messaging, and to improving demand side barriers for both women and men (education, empowerment, employment/ income) to change the environment in which the decisions are being made in order for women to access care [31]. Moreover, attention must be paid to better understand where delays in timely medical intervention occur, which is a significant contributor to maternal and infant mortality and morbidity [33]. Last, more investment is needed in improving the overall quality of maternal and child health services, especially ANC and PNC provision at public healthcare facilities in Chad aimed at reducing maternal and child mortality and morbidity.

In spite of the efforts deployed by the government to improve mobile population’s health in Chad, access to basic care remains a major challenge to most people, due to socioeconomic and geographical reasons [34], but also due internal factors related to cultural practices and norms [24]. The results of this study suggest an increase in vaccination coverage among children of mobile pastoralists as a result of the implementation of joint human and animal vaccination campaigns (One Health). In the thrive towards universal access to health, it will however be important to implement complementary strategies to provide hard-to-reach and marginalized population groups with adequate health services and infrastructures by moving from only mobile outreach campaigns and services to an approach that ensures that these populations visit and access fixed health services [3].

There are several limitations to our study. First, the power calculation of this study was conducted for the initial cross-sectional baseline study with the aim of comparing the use of health services among settled communities and mobile pastoralists across the two districts. The study has therefore limited power to formally test longitudinal hypotheses, and particularly the effectiveness and changes of the PADS programme interventions over time, as no control group was included. Second, most indicators were based on self-reporting which bears the risk to over- or under-estimate the actual utilization ratios [35, 36].

## Conclusion

A combination of health systems strengthening interventions in the health facilities and sensitization campaigns in the communities, including in marginalized and hard-to-reach areas, improved the use of certain maternal and infant health services, especially ANC visits. However, to facilitate equitable access to and use of health care services in particular in times of increased vulnerability (pregnancy) and by certain population groups (mobile pastoralists) in hard-to-reach areas, reinforced health education and culturally adapted communication strategies including gender-specific messaging, will be needed over a sustained period of time.

## Supplementary Information

**Additional file 1: Table S1**. STROBE 2007 checklist of information to include when reporting observational studies. Description of data: The checklist is a quality improvement tool for ensuring that no important information is missed when working on observational studies.

**Additional file 2: Table S2**. English translation of the data collection instrument. Description of data: The file is taken from the ODK version of the questionnaire, originally created in French language, and was translated for the purpose of this publication.

## Data Availability

The datasets used and/or analysed during the current study are not publically available as they were generated in a project context for internal monitoring. The data collection instrument exists in ODK format and has been included as supplemental information (Supplementary material; Table S2). It was initially created in French language and was translated for the purposes of this publication at request of the journal. The French version of the instrument and the datasets are available from the corresponding author on reasonable request.

## Abbreviations

AIDS: Acquired immunodeficiency syndrome
ANC: Antenatal care
CI: Confidence interval
COM: Contrats d’objectifs et de moyen
CSSI: Centre de Support en Santé Internationale
DHS: Demographic and Health Survey
DS: District sanitaire
HIV: Human immunodeficiency virus
ODK: Open data kit
ORs: Odds ratios
PADS: Programme d’appui aux Districts Sanitaires
PNC: Postnatal care
SDC: Swiss Agency for Development and Cooperation
SES: Socio-economic status
Swiss TPH: Swiss Tropical and Public Health Institute

## References

[1] INSEED and ICF International (2016). Enquête Démographique et de Santé et à Indicateurs Multiples au Tchad 2014–2015.

[2] Zinsstag J, Ould Taleb M, Craig PS (2006). Editorial: health of nomadic pastoralists: new approaches towards equity effectiveness. Tropical Med Int Health.

[3] Azétsop J, Ochieng M (2015). The right to health, health systems development and public health policy challenges in Chad. Philos Ethics Humanit Med.

[4] Institute for Health Metrics and Evaluation (2017). Global Burden of Disease compare and visualisation.

[5] MSP (2018). Plan national de développement sanitaire: PNDS3 2018–2021 - Tchad.

[6] OCHA (2020). Chad: humanitarian situation overview - February 2020.

[7] Global Burden of Disease Health Financing Collaborator Network (2019). Past, present, and future of global health financing: a review of development assistance, government, out-of-pocket, and other private spending on health for 195 countries, 1995-2050. Lancet.

[8] Sripad P, Ozawa S, Merritt MW, Jennings L (2018). Exploring meaning and types of Trust in Maternity Care in Peri-urban Kenya: a qualitative cross-perspective analysis. Qual Health Res.

[9] MSP (2019). Disponibilités et capacités opérationnelles des services de santé*.* Rapport de l'enquête SARA.

[10] Lechthaler F, Abakar MF, Schelling E, Hattendorf J (2018). Bottlenecks in the provision of antenatal care: rural settled and mobile pastoralist communities in Chad. Tropical Med Int Health.

[11] Open Data Kit: 2008 17 March 2020]; Available from: https://opendatakit.org/.

[12] Filmer D, Pritchett LH (2001). Estimating wealth effects without expenditure data--or tears: an application to educational enrollments in states of India. Demography.

[13] Winkelmann R, Boes S (2006). Analysis of microdata.

[14] MSP (2020). Annuaire des statistiques sanitaires du Tchad 2018.

[15] Kuhnt J, Vollmer S (2017). Antenatal care services and its implications for vital and health outcomes of children: evidence from 193 surveys in 69 low-income and middle-income countries. BMJ Open.

[16] Mbuagbaw L, Medley N, Darzi AJ, Richardson M (2015). Health system and community level interventions for improving antenatal care coverage and health outcomes. Cochrane Database Syst Rev.

[17] Mbuagbaw LC, Gofin R (2011). A new measurement for optimal antenatal care: determinants and outcomes in Cameroon. Matern Child Health J.

[18] AbouZahr C, Wardlaw T (2003). Antenatal care in developing countries: promises, achievements and missed opportunities-an analysis of trends, levels and differentials, 1990–2001.

[19] Simkhada B, Teijlingen ER, Porter M, Simkhada P (2008). Factors affecting the utilization of antenatal care in developing countries: systematic review of the literature. J Adv Nurs.

[20] Yaya S, Bishwajit G, Ekholuenetale M, Shah V (2017). Timing and adequate attendance of antenatal care visits among women in Ethiopia. J PLoS One.

[21] Moller A-B, Petzold M, Chou D, Say L (2017). Early antenatal care visit: a systematic analysis of regional and global levels and trends of coverage from 1990 to 2013. J The Lancet Global Health.

[22] Kanyangarara M, Munos MK, Walker N (2017). Quality of antenatal care service provision in health facilities across sub-Saharan Africa: evidence from nationally representative health facility assessments. J Glob Health.

[23] Erismann S, Lechthaler F, Gami JP (2018). Evaluation de la qualité des services de santé dans deux districts sanitaires du Tchad: Yao et Danamadji. Rapport de l'étude de fin de la phase 1.

[24] Ali M, Cordero JP, Khan F, Folz R (2019). ‘Leaving no one behind’: a scoping review on the provision of sexual and reproductive health care to nomadic populations. BMC Womens Health.

[25] SwissTPH. Étude sur l'accès et l'utilisation des services de santé maternelle et néonatale dans le district sanitaire de Danamadji. Moyen-Chari; 2016.

[26] Mannava P, Durrant K, Fisher J, Chersich M (2015). Attitudes and behaviours of maternal health care providers in interactions with clients: a systematic review. Glob Health.

[27] Kerber KJ, de Graft-Johnson JE, Bhutta ZA, Okong P (2007). Continuum of care for maternal, newborn, and child health: from slogan to service delivery. Lancet.

[28] Souza JP, Gülmezoglu AM, Vogel J, Carroli G (2013). Moving beyond essential interventions for reduction of maternal mortality (the WHO multicountry survey on maternal and newborn health): a cross-sectional study. Lancet.

[29] Gill K, Pande R, Malhotra A (2007). Women deliver for development. Lancet.

[30] Bhutta ZA, Ali S, Cousens S, Ali TM (2008). Alma-Ata: rebirth and revision 6 interventions to address maternal, newborn, and child survival: what difference can integrated primary health care strategies make?. Lancet.

[31] Elmusharaf K, Byrne E, O'Donovan D (2015). Strategies to increase demand for maternal health services in resource-limited settings: challenges to be addressed. BMC Public Health.

[32] UNFPA and WHO (2009). Mental health aspects of women's reproductive health: a global review of the literature.

[33] Thaddeus S, Maine D (1994). Too far to walk: maternal mortality in context. Soc Sci Med.

[34] MSP (2016). Politique nationale de santé. 2016–2030.

[35] Boerma JT, Black RE, Sommerfelt AE, Rutstein SO (1991). Accuracy and completeness of mothers' recall of diarrhoea occurrence in pre-school children in demographic and health surveys. Int J Epidemiol.

[36] Binyaruka P, Borghi J (2018). Validity of parental recalls to estimate vaccination coverage: evidence from Tanzania. BMC Health Serv Res.

