# Supplementary material for: Effects of a four-year health systems intervention on the use of maternal and infant health services: results from a programme evaluation in two districts of rural Chad
Source: BMC Public Health. 2021 Dec 19;21:2304. doi: 10.1186/s12889-021-12330-2 (PMC8684686; doi:10.1186/s12889-021-12330-2)
Supplement: Supplementary file 1 — Additional file 1: Table S1. STROBE 2007 checklist of information to include when reporting observational studies. Description of data: The checklist is a quality improvement tool for ensuring that no important information is missed when working on observational studies. [file 12889_2021_12330_MOESM1_ESM.docx]

# Table S1: STROBE 2007 checklist of information to include when reporting observational studies

| **Section/Topic** | **Item No** | **Checklist item** | **Reported in Section** |
| --- | --- | --- | --- |
| **Title and abstract** | | | |
|  | 1a | Indicate the study’s design with a commonly used term in the title or the abstract | Abstract (Methods) |
|  | 1b | Provide in the abstract an informative and balanced summary of what was done and what was found | Abstract (Methods and Results) |
| **Introduction** | | | |
| Background/rationale Objectives | 2 | Scientific background and explanation of rationale | Background |
|  | 3 | Specific objectives or hypotheses | Background |
| **Methods** | | | |
| Study design | 4 | Present key elements of study design early in the paper | Methods (Study design and sampling method) |
| Setting | 5 | Describe the setting, locations, and relevant dates, including periods of recruitment, exposure, follow-up, and data collection | Methods (Study setting, study design and sampling method) |
| Participants | 6 | Give the eligibility criteria, and the sources and methods of selection of participants | Methods (Study design and sampling method) |
| Variables | 7 | Clearly define all outcomes, exposures, predictors, potential confounders, and effect modifiers. Give diagnostic criteria, if applicable | Methods (Analysis) |
| Data sources/ measurement | 8 | For each variable of interest, give sources of data and details of methods of assessment (measurement). Describe comparability of assessment methods if there is more than one group | Methods (Analysis) |
| Bias | 9 | Describe any efforts to address potential sources of bias | N/A |
| Study size | 10 | Explain how the study size was arrived at | Methods (Study design and sampling method) |
| Quantitative variables | 11 | Explain how quantitative variables were handled in the analyses. If applicable, describe which groupings were chosen and why | NA |
| Statistical methods | 12 | (a) Describe all statistical methods, including those used to control for confounding | Methods (Analysis), where applicable. |
|  |  | (b) Describe any methods used to examine subgroups and interactions |  |
|  |  | (c) Explain how missing data were addressed |  |
|  |  | (d) If applicable, describe analytical methods taking account of sampling strategy |  |
|  |  | (e) Describe any sensitivity analyses |  |
| **Results** | | | |
| Participants | 13 | (a) Report numbers of individuals at each stage of study—eg numbers potentially eligible, examined for eligibility, confirmed eligible, included in the study, completing follow-up, and analysed | Results (Socio-demographic characteristics) where applicable, Table 1 and Figure 1 |
|  |  | (b) Give reasons for non-participation at each stage |  |
|  |  | (c) Consider use of a flow diagram |  |
| Descriptive data | 14a | (a) Give characteristics of study participants (eg demographic, clinical, social) and information on exposures and potential confounders | Results (Socio-demographic characteristics) and Table 1 |
|  | 14b | (b) Indicate number of participants with missing data for each variable of interest | N/A |
| Outcome data | 15 | Report numbers of outcome events or summary measures | Results, Table 2, 3, 4, 5, 6, 7 and 8 |
| Main results | 16 | (a) Give unadjusted estimates and, if applicable, confounder-adjusted estimates and their precision (eg, 95% confidence interval). Make clear which confounders were adjusted for and why they were included | Results |
|  |  | (b) Report category boundaries when continuous variables were categorized |  |
|  |  | (c) If relevant, consider translating estimates of relative risk into absolute risk for a meaningful time period |  |
| Other analyses | 17 | Report other analyses done—eg analyses of subgroups and interactions, and sensitivity analyses | NA |
| **Discussion** | | | |
| Key results | 18 | Summarise key results with reference to study objectives | Discussion |
| Limitations | 19 | Discuss limitations of the study, taking into account sources of potential bias or imprecision. Discuss both direction and magnitude of any potential bias | Discussion (including limitations) |
| Interpretation | 20 | Give a cautious overall interpretation of results considering objectives, limitations, multiplicity of analyses, results from similar studies, and other relevant evidence | Discussion |
| Generalisability | 21 | Discuss the generalisability (external validity) of the study results | Discussion (including limitations) |
| **Other information** | | |  |
| Funding | 22 | Give the source of funding and the role of the funders for the present study and, if applicable, for the original study on which the present article is based | Declarations (Funding) |
